# Detection of Highly Pathogenic Avian Influenza A(H5N1) Clade 2.3.4.4b Genotype D1.2 Virus in Swine after Experimental Inoculation

**DOI:** 10.3201/eid3208.251765

**Published:** 2026-08

**Authors:** Hannah Seger, Amy L. Baker, Alexandra C. Buckley, Tavis K. Anderson, Alexey Markin, Alessandra Campos, Bruno Caetano Trindade, Marissa Vincent, Giovana Ciacci Zanella, Mia Torchetti, Kristina Lantz, Bailey Arruda

**Affiliations:** Oak Ridge Institute for Science and Education, Oak Ridge, Tennessee, USA (H. Seger); US Department of Agriculture Agricultural Research Service National Animal Disease Center, Ames, Iowa, USA (H. Seger, A.L. Baker, A.C. Buckley, T.K. Anderson, A. Markin, A. Campos, B.C. Trindade, M. Vincent, G.C. Zanella, B. Arruda); Iowa State University College of Veterinary Medicine, Ames (G.C. Zanella); US Department of Agriculture Animal and Plant Health Inspection Service National Veterinary Services Laboratories, Ames (M. Torchetti, K. Lantz)

**Keywords:** influenza, viruses, avian influenza, swine, highly pathogenic avian influenza, influenza A virus, H5N1, United States

## Abstract

Highly pathogenic avian influenza H5NX clade 2.3.4.4b viruses continue to circulate globally. Reintroduction of Eurasian lineage viruses into North America and reassortment with endemic low pathogenicity strains have resulted in new genotypes, including D1.2. To assess pathogenicity and cellular tropism, we intranasally inoculated genotype D1.2 virus into pigs. We isolated virus from nasal secretions from most inoculated animals for multiple days. At 5 days postinoculation, PCR and immunohistochemistry detected virus in musculoskeletal, respiratory, digestive, lymphatic, and nervous systems, and virus was isolated from meat juice. At 35 days postinoculation, we detected viral antigen and low levels of RNA in the brain of an animal with lesions consistent with a viral etiology and found viral antigen in the ethmoid of 2 animals. Consistent detection in nasal swab specimens, combined with subclinical respiratory infection, suggest that identifying infection in commercial swine without overt respiratory signs could be difficult.

Influenza A viruses (IAVs) remain the most common cause of pandemics (*1*). Highly pathogenic avian influenza (HPAI) A virus strains belonging to the goose/Guangdong H5NX lineage (Gs/Gd) have pandemic potential. Continued circulation and reintroductions have resulted in numerous genotypes within H5NX clade 2.3.4.4b viruses because of reassortment with low pathogenicity avian influenza A viruses (LPAIV) (*2*–*5*). In addition, the ecology of currently circulating H5NX clade 2.3.4.4b viruses has become increasingly complex, including continued circulation in a broad range of wild birds, frequent spillover into mammals, and mammal-to-mammal transmission in multiple environments (*5*,*6*). Across those different infected species, clinical disease and pathology are variable. Mammal-to-mammal or avian-to-mammal transmission has led to a subset of strains that contain known mammalian adaptation markers (*7*,*8*).

Swine have historically been considered an intermediary host and mixing vessel for avian influenza viruses that can lead to mammalian adaptation and in which novel viruses with pandemic potential might be generated (*9*). Swine can be naturally infected with avian IAVs , human IAVs, and endemic swine-adapted IAVs and can express sialic acid receptors for both mammalian and avian IAVs in their respiratory epithelium (*10*). The introduction and subsequent reassortment of avian and human IAVs in the swine population have driven the expansion of swine IAV genetic diversity, resulting in all endemic swine IAV in the United States containing human-origin gene segments (*11*,*12*). Whereas host adaptation might require multiple mutations, reassortment of a non–host-adapted IAV and a host-adapted IAV can enable the virus to jump many of those barriers (*13*,*14*).

On October 29, 2024, HPAI H5N1 clade 2.3.4.4b genotype D1.2 was confirmed in 1 sow housed in a backyard animal holding in Oregon, USA (*15*). We sought to assess the pathogenicity and cellular tropism of an HPAI H5N1 strain that was collected from that Oregon farm site in other swine by experimental infection via an intranasal route. The animal study (Appendix 1 Figure 1) was conducted in compliance with the Institutional Animal Care and Use Committee of the US Department of Agriculture Agricultural Research Service National Animal Disease Center under the Biosafety Level 3 guidelines.

## Materials and Methods

### Swine Pathogenesis Study

We inoculated crossbred 4-week-old pigs intranasally by using a mucosal atomization device (Teleflex, https://www.teleflex.com) with 1 mL per nostril of either 10^5.6^ 50% tissue culture infectious dose (TCID_50_)/mL of A/peafowl/Oregon/24-031478-001/2024 (D1.2/OR; n = 11) or phosphate-buffered saline (n = 3). The D1.2/OR isolate was collected from a peafowl on the Oregon farm site at the same time as the sow detection (Appendix 1).

### Phylogenetics and Mammalian Adaptation Markers

We classified D1.2/OR as an HPAI H5N1 clade 2.3.4.4b genotype D1.2 virus by using the GenoFLU pipeline (*16*). To assess the representativeness of D1.2/OR in relation to other H5Nx strains circulating in North America since 2022, we downloaded all segments for H5 strains collected in that period from the National Center for Biotechnology Information Virus database (https://www.ncbi.nlm.nih.gov/labs/virus) (*17*); we downloaded a total of 79,576 segments. We then determined the H5 viruses that had all 8 segments (n = 9,378) and genotyped them by using GenoFLU-multi (https://github.com/moncla-lab/GenoFLU-multi). We used all successfully genotyped viruses in a subsequent phylogenetic analysis of the hemagglutinin (HA) and neuraminidase (NA) segments (Appendix 1). We identified mutations and mammalian adaptation markers in D1.2/OR and H5 viruses with all 8 segments via FluMut without advanced options (*18*).

### Macroscopic and Microscopic Evaluation

At necropsy, we estimated the percentage of purple-red consolidations typical of IAV for each lung lobe and calculated a weighted average, as previously described (*19*). We evaluated multiple sections per tissue type by histopathology (Appendix 1) and manually performed immunohistochemical (IHC) staining by using a rabbit polyclonal anti–influenza A nucleoprotein (NP) primary antibody (GeneTex, https://www.genetex.com), as previously described (*20*). To further support detection of IAV by IHC for the cerebrum of animal 650, we modified the primary antibody concentration from 0.215 µg/mL to 0.113 µg/mL. We performed RNA detection on a Ventana Discovery Ultra (Roche Diagnostics, https://www.roche.com) (Appendix 1).

### Viral Detection by Reverse Transcription PCR and Isolation

We extracted RNA from nasal, fecal, ileal, and spiral colon swab samples and from whole blood in molecular transport medium, serum, oral fluids, bronchoalveolar lavage fluid (BALF), diaphragm meat juice, and tissue samples by using the MagMAX-96 Viral RNA Isolation Kit (Thermo Fisher Scientific, https://www.thermofisher.com). We subjected extracted RNA to real-time reverse transcription PCR (rRT-PCR) by using the VetMAX-Gold SIV Detection Kit (Thermo Fisher Scientific) according to the manufacturer’s recommendations, by which a positive interpreted result has a cycle threshold (Ct) value <38.0 and a suspected result has a Ct value of 38.0–40.0 (Appendix 1). We submitted samples with rRT-PCR and Ct values <35, including nasal swab, BALF, brain, lung, diaphragm tissue, diaphragm meat juice, and spleen samples, to the US Department of Agriculture National Veterinary Services Laboratories for virus isolation, as previously described (Appendix 1) (*20*,*21*).

### Serology

We determined seroconversion by using an IAV NP-blocking ELISA (IDEXX, https://www.idexx.com), for which an optical density of <0.6 at 450 nm was considered positive, and ID Screen Influenza H5 3.0 Multi-Species, Indirect ELISA (Innovative Diagnostics, https://www.innovative-diagnostics.com), for which a signal-to-noise ratio of <38.5% was considered positive, according to the manufacturers’ recommendations. We also performed H5-specific hemagglutination inhibition and serum neutralization (Appendix 1), for which a reciprocal titer >40 was considered positive.

## Results

### Phylogenetics and Mammalian Adaptation Markers

The D1.2/OR HA gene was, on average, 2 aa different from a D1.1 HA gene, and the NA was, on average, 4 aa different from a D1.1 NA gene. D1.2/OR harbored 16 mammalian adaptation markers of H5N1 strains; however, most were conserved across nearly all analyzed strains, except for specific mutations in polymerase basic (PB) 1 (PB1-F2:N66S) and PB2 (L89V, G309D, T339K, R477G, I495V, K627E, A676T) (Appendix 2).

### Subclinical Respiratory and Mild Enteric Signs Observed

We compiled results for the clinical scoring system (Appendix 1 Table 1) into clinical scores (Appendix 1 Table 2). We did not observe respiratory clinical signs or fever in any animal throughout the study (Appendix 1 Tables 2, 3). We observed moderate diarrhea in 1 inoculated animal, starting at 3 DPI through necropsy at 5 DPI. We noted lethargy and diarrhea in the 3 remaining inoculated animals at 7 DPI, resolving at 14 DPI, and anorexia at 7 and 8 DPI. We did not note enteric signs in control animals.

**Table 1:** Nasal swab specimen Ct values for pigs in study of systemic distribution and protracted detection of highly pathogenic avian influenza A(H5N1) clade 2.3.4.4b genotype D1.2 virus in swine after experimental inoculation*

Group and pig ID	DPI									
	0	1	2	3	4	5	6	7	14	35
D1.2/OR, necropsy at 5 DPI										
641	40.0	**33.1**	**25.6**	**29.9**	32.6	**23.6**	–	–	–	–
642	40.0	**30.7**	**22.1**	**29.1**	**32.3**	34.6	–	–	–	–
643	40.0	35.3	**29.4**	37.0	40.0	40.0	–	–	–	–
644	40.0	32.5	31.0	39.8	40.0	38.8	–	–	–	–
645	40.0	**25.0**	**26.6**	**30.7**	**29.1**	**27.9**	–	–	–	–
646	40.0	34.3	34.6	37.0	38.3	40.0	–	–	–	–
647	40.0	**28.9**	**25.2**	31.1	34.4	34.7	–	–	–	–
648	40.0	**28.0**	**26.2**	**29.0**	**32.8**	**28.1**	–	–	–	–
D1.2/OR, necropsy at 35 DPI										
649	40.0	**24.8**	**19.6**	**26.9**	**31.0**	**34.8**	36.5	35.1	39.8	40.0
650	40.0	**32.4**	**26.8**	**32.7**	**33.3**	36.1	36.5	36.6	40.0	40.0
651	40.0	**28.5**	**26.9**	31.3	**32.7**	35.3	36.9	38.1	40.0	40.0
Control, necropsy at 5 DPI										
652	40.0	40.0	40.0	40.0	40.0	40.0	–	–	–	–
653	40.0	40.0	40.0	40.0	40.0	40.0	–	–	–	–
654	40.0	40.0	40.0	40.0	40.0	40.0	–	–	–	–

*Animals were humanely euthanized at 5 or 35 DPI. Samples were tested by quantitative reverse transcription PCR. Light gray shading indicates samples with positive results (Ct <38.0); dark gray shading indicates a suspected result (Ct 38.0–40.0). Samples with a Ct value <35 were selected for virus isolation; bold indicates samples from which virus was isolated. Dashes (–) indicate no sample was taken because the animal had been euthanized. Ct, cycle threshold; DPI, days postinoculation.

### Consistent Viral RNA Detection and Isolation from Nasal Secretions

Samples collected before inoculation from all animals and all samples collected from control animals were negative for IAV by rRT-PCR. However, we detected viral RNA by rRT-PCR in all inoculated animals at 1, 2, and 3 DPI (Table 1). The last detection of IAV RNA in a nasal swab sample was at 14 DPI. We subjected nasal swab samples with a Ct value of <35 to virus isolation and isolated virus from 8 of 10 samples at 1 DPI, 9 of 11 samples at 2 DPI, 6 of 8 samples at 3 DPI, 6 of 8 samples at 4 DPI, and 5 of 6 samples at 5 DPI (Table 1). In total, 34 of 43 nasal swab samples subjected to virus isolation were positive, and 8 of 11 inoculated animals shed infectious virus via nasal secretions over 5 days. Detection of IAV in fecal swab samples was inconsistent; we detected virus in 5 animals with low viral RNA, and most detections (3 of 8) were at 2 DPI (Appendix 1 Table 4). The last detection of IAV RNA in a fecal swab sample was at 14 DPI, within the suspected range. We did not detect viral RNA in blood or serum from any animal but did detect RNA in oral fluids at 1, 2, and 3 DPI (Appendix 1 Table 5).

### Limited Neutralizing Antibody Response at 35 DPI

We did not detect seroconversion in the animals euthanized at 5 DPI by NP or H5 ELISA (Appendix 1 Table 6). We detected seroconversion in the 3 remaining inoculated animals by NP, but not H5 ELISA, at 7 DPI. At 35 DPI, all remaining animals were NP and H5 ELISA positive, 2 animals were positive by serum neutralization, and 1 animal was positive by hemagglutinin inhibition.

### Systemic Detection and Protracted Detection via rRT-PCR and Virus Isolation

At 5 DPI, we detected viral RNA by rRT-PCR in multiple samples of inoculated animals, including BALF, brain, spleen, spiral colon swab, ileal swab, diaphragm meat juice, and diaphragm (Table 2), but not in the pancreas or heart. Of the postmortem samples at 5 DPI, viral RNA was most consistently detected in the brain (6 of 8) and BALF (5 of 8). We also isolated virus from rRT-PCR–positive meat juice samples. At 35 DPI, we detected viral RNA in the brain (1 of 3; animal 650), lung (1 of 3; animal 649), and spiral colon contents (1 of 3; animal 649) within or below the suspected range. We did not detect viral RNA in any sample taken from control animals at necropsy.

**Table 2:** Necropsy Ct values for tissue sample from pigs in study of systemic distribution and protracted detection of highly pathogenic avian influenza A(H5N1) clade 2.3.4.4b genotype D1.2 virus in swine after experimental inoculation*

Group and pig ID	Sample source									
	BALF	Brain	Heart	Lung, homogenized	Diaphragm	Meat juice	Ileum swab	Spiral colon swab	Pancreas	Spleen
D1.2/OR, necropsy at 5 DPI										
641	32.4	30.1	40.0	38.6	37.4	**32.0**	40.0	36.0	40.0	34.7
642	40.0	39.8	40.0	40.0	40.0	40.0	40.0	40.0	40.0	39.1
643	**33.1**	33.6	40.0	37.7	40.0	40.0	40.0	40.0	40.0	40.0
644	40.0	40.0	40.0	40.0	40.0	40.0	40.0	40.0	40.0	40.0
645	**30.6**	36.3	40.0	40.0	38.5	**31.2**	39.2	36.1	40.0	35.0
646	40.0	40.0	40.0	40.0	40.0	40.0	40.0	40.0	40.0	40.0
647	**30.4**	39.8	40.0	**27.5**	40.0	36.4	40.0	40.0	40.0	40.0
648	32.7	35.8	40.0	40.0	40.0	40.0	39.8	40.0	40.0	40.0
D1.2/OR, necropsy at 35 DPI										
649	40.0	40.0	40.0	38.2	40.0	40.0	40.0	37.5	40.0	–
650	40.0	38.4	40.0	40.0	40.0	40.0	40.0	40.0	40.0	–
651	40.0	40.0	40.0	40.0	40.0	40.0	40.0	40.0	40.0	–
Control, necropsy at 5 DPI										
652	40.0	40.0	40.0	40.0	40.0	40.0	40.0	40.0	40.0	40.0
653	40.0	40.0	40.0	40.0	40.0	40.0	40.0	40.0	40.0	40.0
654	40.0	40.0	40.0	40.0	40.0	40.0	40.0	40.0	40.0	40.0

*Animals were humanely euthanized at day 5 or 35 DPI. Tissues or swabs taken at necropsy at 5 or 35 DPI. Samples were tested by quantitative reverse transcription PCR. Light gray shading indicates samples with positive results (Ct <38.0); dark gray shading indicates a suspected result (Ct 38.0–40.0). Samples with a Ct value <35 were selected for virus isolation; bold indicates samples from which virus was isolated. Dashes indicate missing samples. Ct, cycle threshold; DPI, days postinoculation.

### Histologic Lesions and Antigen Detection Confirming Systemic Distribution and Protracted Detection

Macroscopic evaluations of the turbinate, ethmoid, brain, trachea, spleen, pancreas, heart, intestines, and diaphragm were unremarkable at 5 and 35 DPI. Mild macroscopic lung lesions consistent with IAV infection were observed in 3 of 8 inoculated animals at 5 DPI and confirmed by histologic evaluation and IHC (Appendix 1 Table 7, Figure 2; Appendix 2). At 5 DPI, IHC detected NP antigen in multiple tissues and multiple animals, including the turbinate (3 of 7 animals), ethmoid (7 of 8 animals), trachea (2 of 8 animals), lung (3 of 8 animals), olfactory bulb (1 of 8 animals), tracheobronchial lymph node (6 of 7 animals), ileum (1 of 8 animals), and diaphragm (2 of 2 animals) (Appendix 1 Table 7). We did not detect NP antigen in the spleen or spiral colon of the animals with rRT-PCR–positive samples.

**Figure 1:**
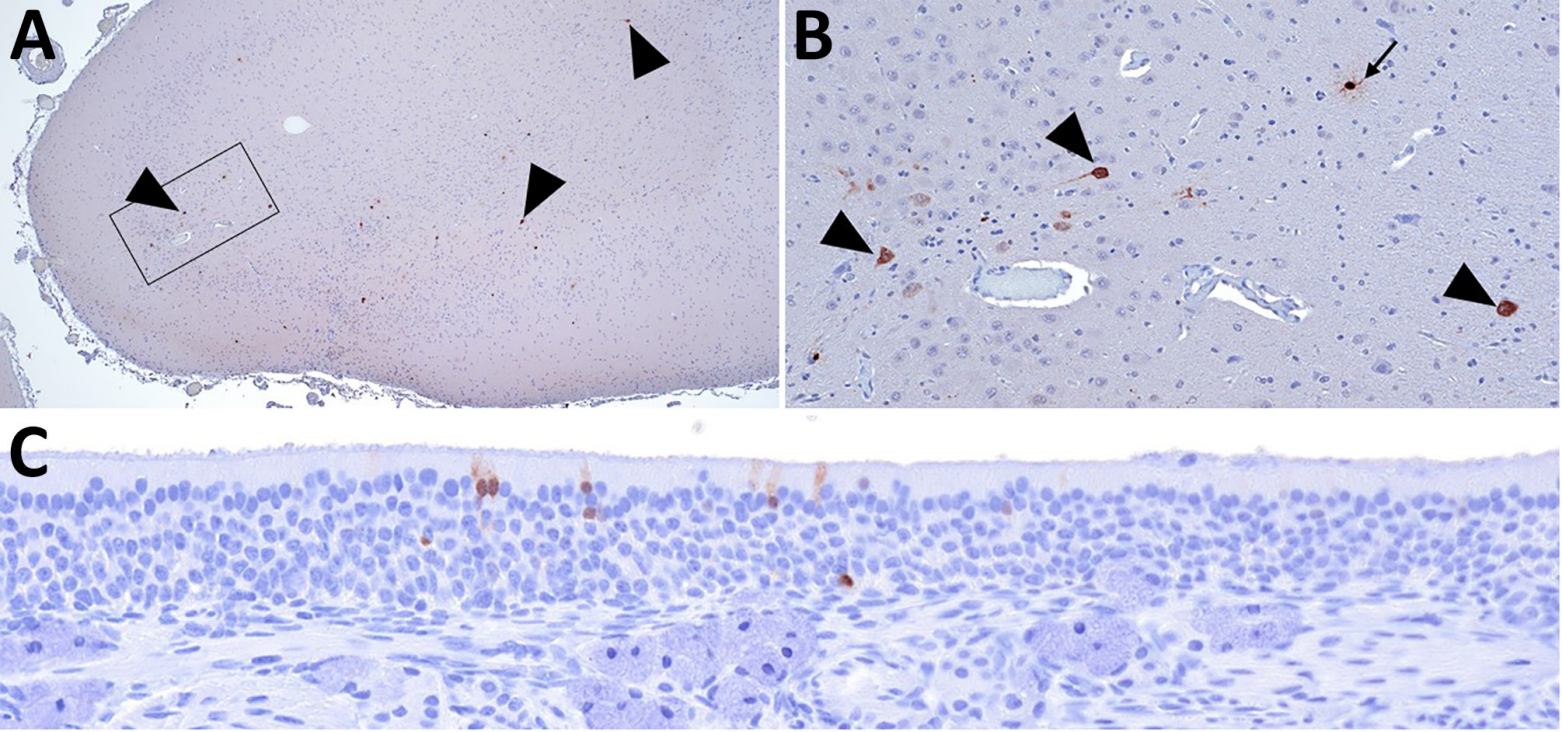
Immunohistochemical stains showing influenza A virus nucleoprotein (NP) antigen detection in pigs at 35 days postinoculation in an area with gliosis in study of systemic distribution and protracted detection of highly pathogenic avian influenza A(H5N1) clade 2.3.4.4b genotype D1.2 virus in swine after experimental inoculation. A) NP antigen within multiple cell types in the cerebrum of animal no. 650 (arrowheads). Original magnification ×40. B) Closer view of boxed area in panel A showing NP antigen within neurons (arrowhead) and glial cell (arrow). Original magnification ×200. C) NP antigen in the cytoplasm and nucleus of the olfactory epithelium of animal no. 649. Original magnification ×200.

We observed histologic lesions within the cerebrum and detected viral RNA by rRT-PCR in 1 of 3 animals at 35 DPI. The observed lesions consisted of occasional small to moderately sized inflammatory infiltrates with co-localization of NP antigen (Figure 1, panels A, B). We also detected NP antigen in the epithelial layer of ethmoids (2 of 2 animals) at 35 DPI (Figure 1, panel C). We did not detect NP antigen in the turbinate, ethmoid, trachea, tracheobronchial lymph node, lung, brain, ileum, or diaphragm of negative control pigs. We also did not detect nonspecific immunolabeling, excluding the primary antibody in the NP IHC–positive diaphragm section of animal 645 or any cerebral sections of animal 650 (Appendix 1 Figure 5, panel A). In situ assays on cerebral tissue from animal 650 at 35 DPI showed IAV NP replicating RNA detected in occasional cells (Appendix 1 Figure 5, panel B) and IAV NP nonreplicating RNA detected in rare cells (Appendix 1 Figure 5, panel C).

### Clade 2.3.4.4b Genotype D1.2 Virus Infecting Different Cell Populations within Different Tissues in Swine

**Figure 2:**
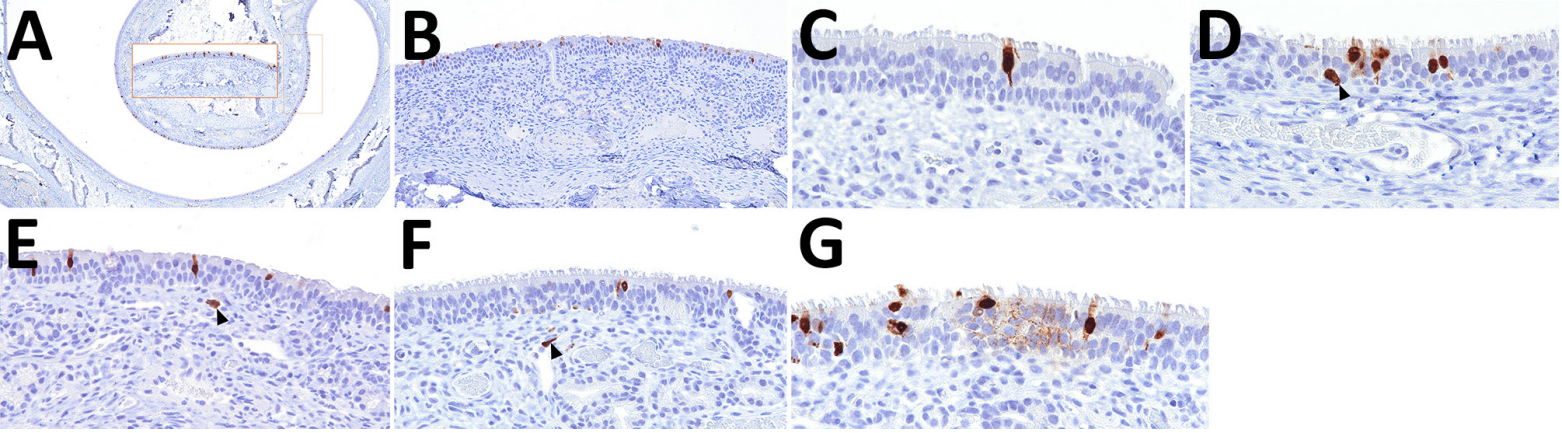
Distribution of influenza A virus nucleoprotein (NP) antigen in the nasal turbinates of pigs in study of systemic distribution and protracted detection of highly pathogenic avian influenza A(H5N1) clade 2.3.4.4b genotype D1.2 virus in swine after experimental inoculation. At 5 days postinoculation, NP antigen (brown) was commonly detected within the epithelial layer without (panel A; animal no. 648; original magnification ×40, inset ×200) and with (panel B; animal no. 642; original magnification ×200) notable loss of cilia of the turbinates, including the nucleus and cytoplasm of ciliated cells (panel C; animal no. 645; original magnification ×600) and less commonly nonciliated cells (panel D, arrowhead; animal no. 648; original magnification ×600). NP was also occasionally found within cells in the submucosa (panels E, F, arrowheads; animal nos. 641 and 648; original magnification ×600) and, rarely, at the cytoplasmic border of an aggregate of cells within the epithelium (panel G; animal no. 648; original magnification ×600).

**Figure 3:**
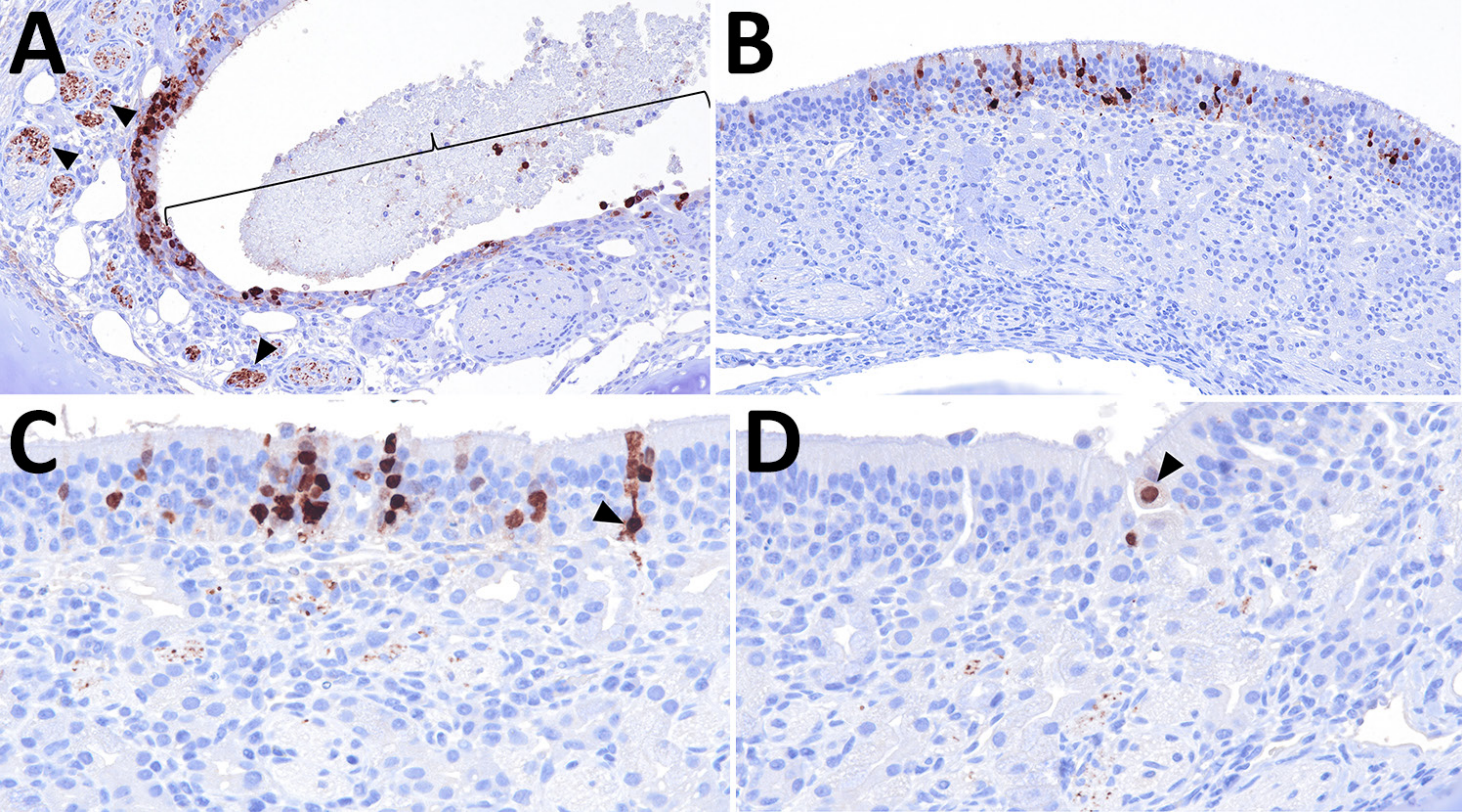
Distribution of influenza A virus nucleoprotein (NP) antigen in the ethmoids of pigs in study of systemic distribution and protracted detection of highly pathogenic avian influenza A(H5N1) clade 2.3.4.4b genotype D1.2 virus in swine after experimental inoculation. At 5 days postinoculation, NP antigen (brown) was detected within the epithelial layer in areas of erosion (brace) and fila olfactoria (panel A, arrowheads; animal no. 641; original magnification ×200) and without (panel B; animal no. 647; original magnification ×200) notable loss of cilia of the ethmoid. Immunolabeling was detected in cells with morphologies consistent with olfactory sensory neurons (panel C; animal no. 642; original magnification ×400) and, rarely, the epithelium of Bowman’s gland (panel D; animal no. 642; original magnification ×400).

We detected NP antigen across multiple tissue types and cell populations within the upper respiratory tract by IHC. NP IAV antigen commonly appeared within the epithelial layer with and without notable loss of cilia of the turbinate, including the nucleus and cytoplasm of ciliated cells, less commonly in nonciliated cells, occasionally within cells in the submucosa, and rarely at the cytoplasmic border of an aggregate of cells within the epithelium (Figure 2). Similarly, NP IAV antigen occurred within the epithelial layer with and without notable loss of cilia of the ethmoid (Figure 3). We found immunolabeling in varying cell morphologies consistent with olfactory sensory neurons and at varying levels of the ethmoid, including the fila olfactoria and, rarely, the epithelium of Bowman’s gland (Figure 3).

**Figure 4:**
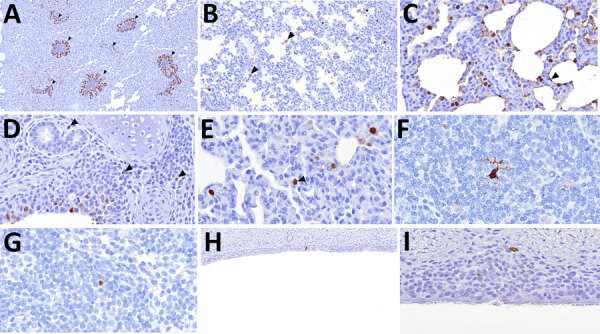
Distribution of influenza A virus nucleoprotein (NP) antigen in the lower respiratory tract and tracheobronchial lymph node of pigs in study of systemic distribution and protracted detection of highly pathogenic avian influenza A(H5N1) clade 2.3.4.4b genotype D1.2 virus in swine after experimental inoculation. At 5 days postinoculation, NP antigen (brown) was detected within the lower respiratory tract and associated lymphoid tissue. Immunolabeling in the lung was detected in the epithelium of conducting airways (panel A, arrowheads; animal no. 647; original magnification ×200), pulmonary alveolar macrophages (panel B; animal no. 645; original magnification ×200) lining the alveolar septa (arrowhead) and type II pneumocytes (brace), probable leukocytes within the bronchus-associated lymphoid tissue (panel D, arrowheads; animal no. 648; original magnification ×400), and leukocytes within the alveolar septa (panel E, arrowhead; animal no. 646; original magnification ×600). Viral antigen was also detected in the nucleus of leukocytes with morphologies consistent with an antigen-presenting leukocyte (panel F; animal no. 645; original magnification ×400) and lymphocyte (panel G; animal no. 646; original magnification ×600) in the tracheobronchial lymph node. NP antigen was only occasionally detected in the epithelial (panel H; animal no. 645; original magnification ×400) and submucosal (panel I; animal no. 643; original magnification ×600) layers of the trachea.

We detected NP antigen across cell populations within the lower respiratory tract and associated lymphoid tissue. Immunolabeling in the lung occurred in the epithelium of conducting airways, lining the alveolar septa, pulmonary alveolar macrophages, leukocytes within the bronchus-associated lymphoid tissue, type II pneumocytes, and probable leukocytes within the alveolar septa (Figure 4, panels A–E). Nuclear staining was present across those cell populations in the lung. We detected viral antigen in the nucleus of leukocytes with morphologies consistent with an antigen-presenting cell and lymphocyte in the tracheobronchial lymph node (Figure 4, panels F, G). NP antigen occasionally appeared in the epithelial and submucosal layer of the trachea (Figure 4, panels H, I).

**Figure 5:**
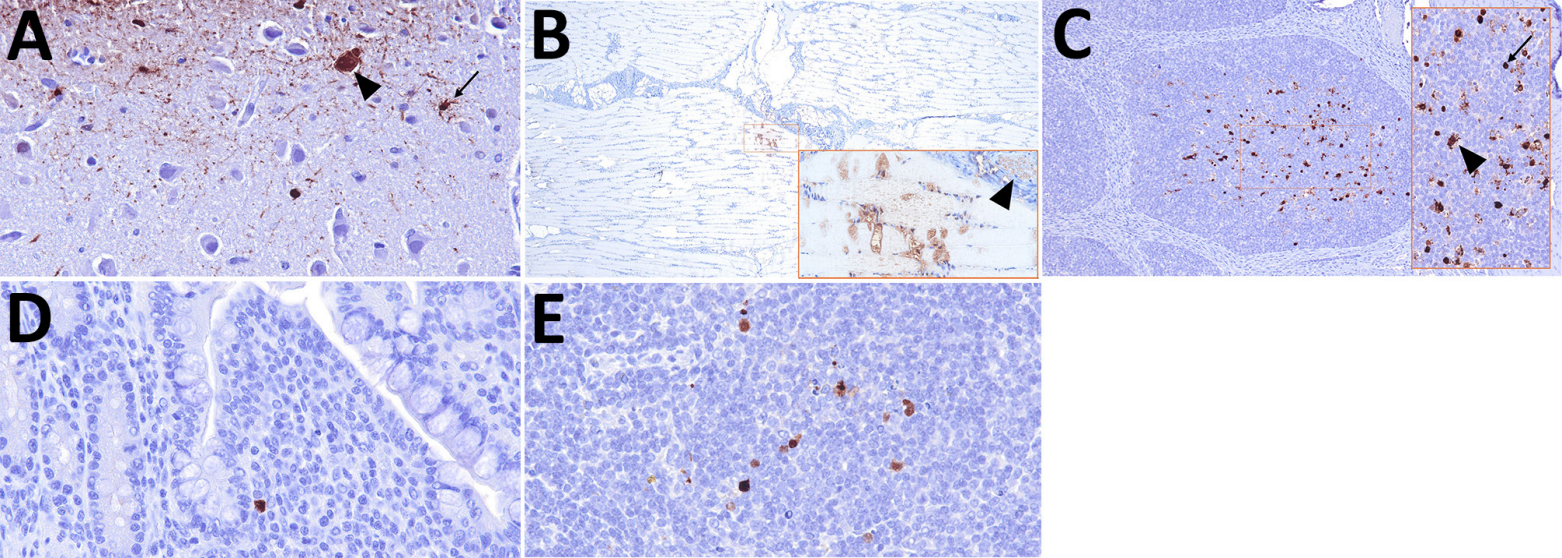
Distribution of influenza A virus nucleoprotein (NP) antigen across multiple body systems in a study of systemic distribution and protracted detection of highly pathogenic avian influenza A(H5N1) clade 2.3.4.4b genotype D1.2 virus in swine after experimental inoculation. NP antigen (brown) was detected at 5 days postinoculation in the neurons (arrowhead) and associated axons (arrows) of the olfactory bulb in the absence of notable lesions (panel A; animal no. 641; original magnification ×400), skeletal myocytes and an adjacent axon (arrowhead) (panel B; animal no. 645; original magnification ×40 and ×400 [inset]), the Peyer’s patch of the ileum in cell morphologies consistent with antigen-presenting leukocytes (arrowhead) and lymphocytes (arrow) (panel C; animal no. 641; original magnification ×100 and ×400 [inset]), and rarely in the lamina propria of the ileum (panel D; animal no. 641; original magnification ×400) and mesenteric lymph node attached to the spiral colon (panel E; animal no. 641; original magnification ×400).

We also detected NP IAV antigen in tissues in close and distant approximation to the respiratory tract. Immunolabeling was present in the neurons and associated axons of the olfactory bulb at 5 DPI and in the neurons of the cerebrum at 35 DPI (Figure 1, panel B; Figure 5, panel A). For the 2 animals in which the virus was isolated from the meat juice of the diaphragm, we detected IAV NP antigen in skeletal myocytes of the diaphragm in both animals and an axon adjacent to the immunolabeled skeletal myocytes in 1 of the animals (Figure 5, panel B). Immunolabeling was also present at 5 DPI in the Peyer’s patch (PP) and rarely in the lamina propria of the ileum and mesenteric lymph node attached to the spiral colon (Figure 5, panels C–E). IHC did not detect antigen in enterocytes or colonocytes. Nuclear staining was seen across different cell morphologies, including those consistent with antigen-presenting cells and lymphocytes.

## Discussion

The ongoing evolution of Gs/Gd lineage H5 viruses has given rise to multiple HA clades, including 2.3.4.4b, which continues to circulate in migratory birds on a global scale (*23*). Subsequent reassortment between HPAI clade 2.3.4.4b virus and LPAIV has produced multiple genotypes, including D1.2, identified in a sow from a backyard menagerie in the United States (*1*,*19*). This genotype is a reassortment of PB2, NP, and NA, and polymerase acidic genes and differs by only 1 gene segment, polymerase acidic, from the D1.1 genotype, which became predominant in birds across all 4 flyways by the end of 2024 (*24*). Here, we demonstrate persistent detection in nasal swab samples, systemic distribution, including detection of HPAI virus in the skeletal muscle of a livestock species, and protracted detection of an HPAI H5N1 clade 2.3.4.4b genotype D1.2 virus in conventional swine.

Phylogenetic and mammalian adaptation marker analyses demonstrated minor differences between the D1.2/OR and D1.1 strains, along with the presence of established mammalian adaptation markers. D1.2/OR clustered within the D1.1 lineage in both the HA and NA phylogenetic trees. The D1.2/OR HA gene was, on average, 2 aa different from a D1.1 HA gene, and the NA was, on average, 4 aa different from a D1.1 NA gene. D1.2/OR harbors established mammalian adaptation markers of H5N1 viruses including the HA mutation T156A, which has been linked to enhanced transmission in guinea pigs and increased affinity for α-2,6-linked sialic acid receptors (*25*,*26*). However, the marker is present in nearly all H5 strains included in this analysis. Mammalian markers not highly conserved across analyzed strains included PB1-F2 (N66S; ≈60%) and PB2 (L89V, G309D, T339K, R477G, I495V, K627E, A676T; ≈60%) mutations. PB1-F2 is an accessory protein that is not required for replication but modulates virulence, apoptosis, and innate immune responses, increasing virulence in mice models when N66S is present and leading to systemic spread in ducks (*27*–*30*). Mammalian markers in PB2 enhance replication by improving polymerase activity, nuclear import, and compatibility with mammalian host proteins. The multiple PB2 mutations observed in D1.2/OR could be considered compensatory for the classical mammalian adaptation marker E627K; that particular constellation of polymorphisms has been associated with increased polymerase activity, replication in mammalian cells, and virulence in mice when 627E was retained (*31*).

The increased geographic dissemination of Gs/Gd viruses has been attributed to their wide host range, effective transmission, reassortment, and adaptation (*1*). The findings in this study of protracted detection at 35 DPI in the respiratory tract (2 of 3 animals) and central nervous system (CNS) (1 of 3 animals) and weak to absent or quickly waning neutralizing antibody response despite consistent nasal detection and systemic spread could also be possible mechanisms contributing to the success of this pathogen at the individual animal level or moderate replication in an atypical host species. Whereas the quantity of viral RNA as determined by rRT-PCR at both 5 and 35 DPI would suggest minimal virus presence, isolation was possible from diaphragm meat juice (Ct values 31.2 and 32.0), and viral antigen was detected by IHC in the diaphragm at 5 DPI (Ct value 38.5) and ethmoid and brain (Ct value 38.4) at 35 DPI.

We documented evidence of replication in multiple tissues and detection and viral isolation in nasal secretions of all intranasally (2 mL) inoculated pigs across multiple days. Other studies in swine have reported inconsistent detection in nasal swab specimens, limited detection outside the respiratory tract, and variable transmission (*20*,*34*; H. Feldmann et al., unpub. data, https://doi.org/10.21203/rs.3.rs-6567595/v1). The consistent detection in nasal secretions, broader viral distribution within tissues, and protracted detection of low viral levels in 2 of 3 animals necropsied at 35 DPI documented in this work compared with the other studies might reflect study design, host, strain, or a combination of those. The more consistent detection in nasal secretions, turbinate and ethmoid compared with the trachea and lung within this study could suggest other viral mechanisms of host and tissue tropism beyond α-2,3 and α-2,6 sialic acid distribution that have not yet been characterized in swine (*35*).

Influenza is frequently associated with intestinal disorders (*36*), but the underlying mechanisms remain elusive. In this study, we observed enteric clinical signs; however, unlike detection in nasal secretions, fecal detection was inconsistent. The primary source of viral RNA in feces is unknown but could be multifaceted (*37*,*38*). Seasonal IAV RNA has been detected in stool samples from patients with respiratory and enteric signs, and HPAI H5N1 virus has been detected in feces and intestinal mucosa of humans (*39*,*40*). We did not detect lesions consistent with atrophic enteritis or NP antigen in enterocytes or colonocytes. The detection of virus within PP may be a result of the PP being a permissive gateway in the gut, enabling antigen sampling by leukocytes at the gut lumen. Expanded evaluation of the enteric system could provide a more refined understanding of the mechanisms involved in the development of enteric signs during influenza.

We detected replicating HPAIV as determined by the presence of nuclear staining via NP IHC in multiple cell types, including leukocytes of varying morphologies in the PP of the ileum, lymphocytes in the lamina propria, and tracheobronchial lymph nodes. Studies have established a variable susceptibility of subsets of antigen-presenting cells to IAV infection (*41*–*44*). Lymphocytes are susceptible to IAV infection (*45*), and infected CD4+ and CD8+ lymphocytes can serve as infection foci for other cells (*42*,*45*). We rarely detected NP antigen at the cytoplasmic borders of a cluster of cells in the upper respiratory tract. That staining pattern is not well documented but could result from tunneling nanotube formation in vivo, with possible implications for reassortment (*46*). The role of immune cells that support viral replication and a rare but interesting staining pattern of cytoplasmic borders in the immune response, viral dissemination, and reassortment warrant further investigation.

The routes by which HPAI H5 viruses disseminate through hosts include neuroinvasion through the olfactory and respiratory pathways (*47*). HPAI H5Nx viruses are thought to use the olfactory, trigeminal, facial, vestibulocochlear, vagus, and upper thoracic sympathetic nerves to enter the CNS of mammals (*47*). The ability to use those nerves to invade the CNS depends on the multibasic cleavage site of HPAI H5 (*47*). Here, we identified the ability of HPAI H5N1 clade 2.3.4.4b genotype D1.2 virus to infect both the CNS and peripheral nervous system in swine. The detection of viral antigen in the fila olfactoria of swine and the olfactory bulb is consistent with the olfactory pathway of neuroinvasion (*47*). The concurrent detection of viral antigen in the diaphragm and adjacent axon as seen in this study supports viral dissemination through the phrenic nerve, a peripheral nerve, in swine and poses a possible public health concern, given that the virus could be isolated from the meat juice of the diaphragm. Additional investigations into the distribution of the virus in skeletal muscles, including those of the head, are warranted.

In conclusion, the intercontinental circulation of HPAI H5Nx viruses of the Gs/Gd lineage is a historic occurrence that has resulted in the infection of many avian and mammalian species with variable clinical manifestations, ranging from subclinical infections to mass mortality events. Host responses to HPAI infection, expression of clinical disease, and associated pathology vary depending on numerous interactions including the host, route of infection, dose, day postinfection, and virus strain (*1*). In this study, we observed no apparent respiratory or systemic signs and minimal neutralizing antibody response, despite consistent detection in nasal swab specimens and systemic distribution including skeletal muscle in inoculated animals. Our data raise concerns over our ability to identify infection in commercial swine that do not exhibit overt respiratory signs while also exhibiting minimal neutralizing antibody response in affected animals. The apparent increased fitness of clade 2.3.4.4b H5Nx viruses and their reassortants in swine raises concerns over public health risks and highlights the need to clarify mammalian adaption and reassortment potential and supports the need for continued surveillance.

Appendix 1 Additional materials and methods for systemic distribution and protracted detection of highly pathogenic avian influenza A(H5N1) clade 2.3.4.4b genotype D1.2 in swine after experimental inoculation.

Appendix 2 Additional data for systemic distribution and protracted detection of highly pathogenic avian influenza A(H5N1) clade 2.3.4.4b genotype D1.2 in swine after experimental inoculation.
