# Supplementary material for: Detection of Highly Pathogenic Avian Influenza A(H5N1) Clade 2.3.4.4b Genotype D1.2 Virus in Swine after Experimental Inoculation
Source: Emerg Infect Dis. 2026 Aug;32(8):1264–74. doi: 10.3201/eid3208.251765 (PMC13426851; doi:10.3201/eid3208.251765)
Supplement: Appendix 1 — Additional materials and methods for systemic distribution and protracted detection of highly pathogenic avian influenza A(H5N1) clade 2.3.4.4b genotype D1.2 in swine after experimental inoculation. [file 25-1765-Techapp-s1.pdf]

Article DOI: https://doi.org/10.3201/eid3208.251765

EID cannot ensure accessibility for supplementary materials supplied by authors. Readers who have difficulty accessing supplementary content should contact the authors for assistance.

Detection of Highly Pathogenic Avian Influenza A(H5N1) Clade 2.3.4.4b Genotype D1.2 in Swine after Experimental Inoculation

Appendix 1

Additional Material and Methods

Swine Pathogenesis Study

Eight D1.2/OR inoculated animals and all sham-inoculated negative controls were necropsied on 5 days post-inoculation (DPI). The remaining three D1.2/OR inoculated animals were necropsied on 35 DPI to assess seroconversion. Bronchoalveolar lavage fluid (BALF), fresh and formalin-fixed tissues for RT-qPCR and microscopic evaluation were collected (Table 2; Appendix 1 Table 7; Appendix 2 Table 1, https://wwwnc.cdc.gov/EID/article/32/8/25-1765-App2.xlsx). A section of fresh diaphragm was frozen at –80 °C, thawed, and meat juice, fluid recovered from muscle after one or more freeze-thaw cycles, was collected. Formalin-fixed tissues were processed routinely.

Fourteen crossbred, 4-week-old pigs free of IAV, porcine reproductive and respiratory syndrome virus, and *Mycoplasma hyopneumoniae* were used in this study. Pigs were confirmed seronegative to IAV by a blocking ELISA (IDEXX) prior to challenge. Animals were observed for changes in behavior, respiratory rate and effort, nasal and ocular discharge, and fecal consistency and given clinical scores (Appendix 1 Table 1). In addition, temperature was measured daily via an implanted thermal microchip (MERCK Animal Health) (Appendix 1 Table 3). Clinical samples included nasal swabs (Copan, no. 503CS01), fecal swabs (Copan, no. 502CS01), blood, and group-level oral fluids. Nasal and fecal swabs were collected on days 0, 1, 2, 3, 4, 5, 6, 7, 14, and 35 DPI and placed in 2 mL of minimum essential medium (MEM). Whole

blood and serum were collected on days 0, 5, 7, 35 DPI. Whole blood was placed into serum separator tubes and in molecular transport media (0.5 mL into 1.5 mL of PrimeStore MTM, Longhorn, Bethesda, MD). Oral fluids were collected daily from -4, 0-4, and 28 DPI from each group with 5/8-inch cotton ropes hung for approximately 1 hour. At necropsy, the skull was longitudinally split caudal to the ethmoids to avoid contamination of brain samples.

Phylogenetics and Mammalian Adaptation Markers

For HA and NA, the sequences were aligned with MAFFT v7.475 (*1*) and phylogenetic trees were constructed using IQ-Tree v2.3.2 (*2*) under the generalized time-reversible substitution model with empirical base frequencies and five free rate categories (*3*). The trees were mid-point rooted, and the branches of the tree were monophyletically colored respective to the inferred genotype using smot v.0.16.0 (*4*) for all major genotypes with at least 5% of detections in the dataset (Appendix 1 Figure 3). The peafowl strain was compared to an average D1.1 strain across the HA and the NA genes using a custom python script (HammerTime, https://github.com/flu-crew/gallimaufry/).

Macroscopic and Microscopic Evaluation

Multiple sections per tissue type were evaluated by histopathology: lung (3- left cranial, right cranial, caudal), brain (Appendix 1 Figure 4), trachea (3 rings), turbinate (3-5 scrolls), and ethmoid (*2*). Due to viral RNA detection by RT-qPCR in the diaphragm of two D1.2 inoculated animals, frozen sections of diaphragm from 641, 645, and a negative control, animal 654, were thawed in 10% neutral buffered formalin at 4°C for 48 hours and then transferred to 70% ethanol and processed routinely. Three or four blocks of diaphragm each containing 2 to 3 sections were cut and processed.

IHC was performed on sections of any tissue or tissue representative swab that was positive by RT-qPCR as well as all brain, lung, trachea, turbinate, ethmoid, and tracheobronchial lymph node sections of all inoculated animals and a tissue representative from a negative control animal. For each run, lung from an IAV infected pig was used as a positive control. A no-primary antibody control was also run on the diaphragm of animal 641 and each brain section of animal 650. A no-primary antibody control was also run on the diaphragm of animal 641. To further support the detection of IAV in the cerebrum in animal 650 by IHC, we further optimized

our assay specifically for this tissue by increasing the primary antibody concentration from 0.215 ug/mL to 0.113 ug/mL and by running no-primary controls concurrently for each slide.

RNAScope detection for replicating and nonreplicating probes was performed on formalin-fixed, paraffin embedded 4-µm sections on a Ventana Discovery Ultra (Roche Diagnostics). Dewaxing, and rehydration were performed on board, and epitope retrieval was performed using Ventana ULTRA Cell Conditioning Solution 2 (Roche, 05279798001) at 95◦C for 64 min. MRNA AP detection was applied (Roche, 322040) followed by AP-PretreatPro (ACD, 322035). Probe, either Replicating: V-influenza-H1N1-H5N1-NP-sense-C1 (biotechne, 1132489) or Non-replicating: V-influenza-H5N1-NP-O1 (biotechne, 504149), was applied. Hybridization was done with VS Universal AP Standard Reagents (Roche, 322040). Counterstaining was performed using hematoxylin (Roche, 760-2021) followed by dehydration and cover slipping. Sections were examined by a veterinary pathologist using an Olympus BX43 light microscope. Photomicrographs were taken using an Olympus DP28 camera. Control probes used include Ss-UBC 2.5 VS (biotechne, 400649 and DapB 2.5 VS (biotechne, 312039).

Viral Detection by RT-qPCR

RNA was extracted from nasal, fecal, ileal, and spiral colon swabs, whole blood in MTM, serum, oral fluids, BALF, meat juice and tissue samples using the MagMAX-TM-96 Viral RNA Isolation Kit per manufacturer's recommendations (ThermoFisher, Catalog #AM1836 (50µl) and #AM1839 (tissue only, 100mg, spin procedure)). Extracted RNA was subjected to real-time reverse transcription PCR (RT-qPCR) and interpreted using the VetMAX-Gold SIV Detection kit per manufacturer's instructions (Life Technologies, Catalog #4415200), a sample with a positive interpreted result by RT-qPCR has a Ct less than 38.0, and a suspect result by RT-qPCR has a Ct between 38.0-40.0.

Serology

H5 specific hemagglutination inhibition (HI) was conducted as described previously (H5N1; rgA/bald 200 eagle/FL/22; provided by Dr. Richard Webby, St. Jude Children's Research Hospital and A/D1.2/ORegon/24-031478-001/2024) (*5*). Prior to HI, serum samples were heat inactivated at 56° C for 30 minutes, treated with receptor destroying enzyme (Hardy Diagnostics, Santa Maria, CA), and adsorbed with 100% turkey red blood cells for 60 minutes to remove nonspecific hemagglutinin inhibitors and natural serum agglutinins. Serum neutralization

was performed as described previously with slight modification (primary antibody 1:250; A/D1.2/Oregon/24-031478-001/20240) (*6*).

Virus Isolation

Samples with RT-qPCR and Ct values at or below 35 including: nasal swabs, BALF, brain, lung, diaphragm tissue, diaphragm meat juice, and spleen were submitted to USDA National Veterinary Services Laboratories (NVSL) and subjected to virus isolation (*7*,*8*). Diaphragm meat juice samples with positive RT-qPCR and CT values below 35 were thawed and briefly vortexed. 0.1 mL of meat juice (undiluted, animal 645; 1:10 dilution in infection media containing TPCK-Trypsin (1:1000), animal 641) was then plated on MDCK-London cells in 48 well tissue culture dish, incubated (48 h, 37 °C, 5% CO2) and subsequently fixed with 4% phosphate-buffered formalin and stained using immunocytochemistry with an anti-IAV nucleoprotein monoclonal antibody as previously described (*7*).

References

1. Katoh K, Standley DM. MAFFT multiple sequence alignment software version 7: improvements in performance and usability. Mol Biol Evol. 2013;30:772–80. PubMed https://doi.org/10.1093/molbev/mst010

2. Minh BQ, Schmidt HA, Chernomor O, Schrempf D, Woodhams MD, von Haeseler A, et al. IQ-TREE 2: New Models and Efficient Methods for Phylogenetic Inference in the Genomic Era. Mol Biol Evol. 2020;37:1530–4. PubMed https://doi.org/10.1093/molbev/msaa015

3. Soubrier J, Steel M, Lee MSY, Der Sarkissian C, Guindon S, Ho SY, et al. The influence of rate heterogeneity among sites on the time dependence of molecular rates. Mol Biol Evol. 2012;29:3345–58. PubMed https://doi.org/10.1093/molbev/mss140

4. Arendsee ZW, Baker ALV, Anderson TK. smot: A python package and CLI tool for contextual phylogenetic subsampling. J Open Source Softw. 2022;7:4193. https://doi.org/10.21105/joss.04193

5. Kitikoon P, Gauger PC, Vincent AL. Hemagglutinin inhibition assay with swine sera. Methods Mol Biol. 2014;1161:295–301. PubMed https://doi.org/10.1007/978-1-4939-0758-8_24

6. Gauger PC, Vincent AL. Serum Virus Neutralization Assay for Detection and Quantitation of Serum-Neutralizing Antibodies to Influenza A Virus in Swine. In: Spackman E, editor. Animal Influenza

Virus. Methods in Molecular Biology, vol. 1161. New York: Humana Press; 2014. https://doi.org/10.1007/978-1-4939-0758-8_26.

7. Kitikoon P, Nilubol D, Erickson BJ, Janke BH, Hoover TC, Sornsen SA, et al. The immune response and maternal antibody interference to a heterologous H1N1 swine influenza virus infection following vaccination. Vet Immunol Immunopathol. 2006;112:117–28. PubMed https://doi.org/10.1016/j.vetimm.2006.02.008

8. Baker AL, Arruda B, Palmer MV, Boggiatto P, Sarlo Davila K, Buckley A, et al. Dairy cows inoculated with highly pathogenic avian influenza virus H5N1. Nature. 2025;637:913–20. PubMed https://doi.org/10.1038/s41586-024-08166-6

Appendix 1 Table 1. Clinical Score Used to Evaluate Animals

Score	Behavior	Respiratory Signs	Cough	Nasal/Ocular Discharge	Diarrhea
0	Normal	Normal	Normal	Normal	Normal
1	Mild lethargy with decrease in ambulation and attitude compared to pen mates	Slightly increased respiratory effort/ slight dyspnea	Occasional cough	Mild increase in rhinorrhea and/or ocular discharge	Maintains some form but is soft - forms a puddle
2	Moderate lethargy with stimulation needed to provoke ambulation	Notable increase in respiratory effort/ dyspnea	Consistent cough	Moderate increase in rhinorrhea and/or conjunctivitis suggestive of an upper respiratory tract infection	Lacks form but still has substance
3	Marked lethargy with ambulation not provoked by stimulation	Severe dyspnea with respiratory distress and tachypnea	Abundant cough	Marked increase in rhinorrhea and/or conjunctivitis with swelling/erythema of the eyelid	Liquid

Appendix 1 Table 2. Clinical Score by Animal and Day Post Inoculation*

	Animal	Day Post Inoculation														
Group	ID	0	1	2	3	4	5	6	7	8	9	10	11	12	13	14
Control	652	0	0	0	0	0	0	-	-	-	-	-	-	-	-	-
	653	0	0	0	0	0	0	-	-	-	-	-	-	-	-	-
	654	0	0	0	0	0	0	-	-	-	-	-	-	-	-	-
D1.2/OR	641	0	0	0	0	0	0	-	-	-	-	-	-	-	-	-
	642	0	0	0	0	0	0	-	-	-	-	-	-	-	-	-
	643	0	0	0	0	0	0	-	-	-	-	-	-	-	-	-
	644	0	0	2	1	1	1	-	-	-	-	-	-	-	-	-
	645	0	0	0	0	0	0	-	-	-	-	-	-	-	-	-
	646	0	0	0	0	0	0	-	-	-	-	-	-	-	-	-
	647	0	0	0	0	0	0	-	-	-	-	-	-	-	-	-
	648	0	0	0	0	0	0	-	-	-	-	-	-	-	-	-
	649	0	0	0	0	0	0	0	4	4	2	1	0	1	0.5	0
	650	0	0	0	0	0	0	0	4	4	2	1	0	1	0.5	0
	651	0	0	0	0	0	0	0	2.5	2	0.5	1	0	0	0	0

*Clinical scores from study animals from 0 to 7 Day Post Inoculation (DPI). Scores for individual animals are the total from all categories (Behavior, Respiratory Signs, Cough, Nasal/Ocular Discharge, and Diarrhea). -, indicates score not taken as animals had been euthanized.

Appendix 1 Table 3. Body Temperature by Animal and Day Post Inoculation*

Group	Animal ID	Day Post Inoculation								
		-1	0	1	2	3	4	5	6	7
Control	652	39.9	40.2	40.1	39.5	39.2	39.5	39.1	-	-
	653	39.8	39.9	40.1	39.5	39.9	38.9	39.8	-	-
	654	39.8	39.7	39.4	39.4	39.5	39.0	39.2	-	-
	641	39.2	39.3	39.7	39.4	39.2	39.2	40.2	-	-
D1.2/OR	642	39.4	39.0	39.7	39.3	38.9	38.8	39.3	-	-
	643	39.4	39.2	39.5	39.4	39.1	39.0	39.2	-	-
	644	39.4	39.4	39.7	39.5	39.2	39.1	39.3	-	-
	645	39.1	39.1	39.3	38.5	38.6	39.0	38.9	-	-
	646	39.2	38.9	39.3	39.1	38.5	38.8	39.2	-	-
	647	39.3	39.5	39.5	39.2	39.1	39.3	39.9	-	-
	648	39.1	39.0	39.2	39.3	39.0	38.3	38.8	-	-
	649	39.3	39.4	39.4	39.1	38.8	39.0	39.0	39.1	39.3
	650	39.1	39.0	39.1	39.1	38.9	38.4	38.5	39.1	39.8
	651	39.0	39.1	39.0	39.4	39.0	38.6	38.9	39.2	39.4

*Body temperature in degrees Celsius (°C) from study animals from 0 to 7 Day Post Inoculation (DPI). Temperature was taken by an implanted thermal microchip (MEREK Animal Health). -, indicates temperature not taken as animals had been euthanized.

Appendix 1 Table 4. Fecal Swab RT-qPCR Cycle Threshold Values*

Group	Animal ID	Day Post Inoculation								
		0	1	2	3	4	5	6	7	14	35
D1.2/OR	641	40.0	40.0	40.0	0.0	40.0	35.8	-	-	-	-
	642	40.0	40.0	35.4	40.0	40.0	40.0	-	-	-	-
	643	40.0	39.4	38.2	40.0	40.0	40.0	-	-	-	-
	644	40.0	40.0	40.0	40.0	40.0	40.0	-	-	-	-
	645	40.0	40.0	40.0	40.0	40.0	40.0	-	-	-	-
	646	40.0	40.0	40.0	40.0	40.0	40.0	-	-	-	-
	647	40.0	40.0	40.0	40.0	40.0	40.0	-	-	-	-
	648	40.0	40.0	40.0	40.0	40.0	40.0	-	-	-	-
	649	40.0	40.0	38.5	36.7	40.0	40.0	40.0	40.0	39.8	40.0
	650	40.0	40.0	40.0	40.0	40.0	40.0	40.0	40.0	40.0	40.0
	651	40.0	40.0	40.0	38.0	40.0	40.0	40.0	39.7	40.0	40.0
Control	652	40.0	40.0	40.0	40.0	40.0	40.0	-	-	-	-
	653	40.0	40.0	40.0	40.0	40.0	40.0	-	-	-	-
	654	40.0	40.0	40.0	40.0	40.0	40.0	-	-	-	-

*Blue highlighted values indicate a sample with a positive interpreted result by RT-qPCR per the manufacture's recommendations (Ct <38) and orange highlighted values indicate a suspect result by RT-qPCR (Ct values between 38.0-40.0). -, indicates sample not taken as animals had been euthanized.

Appendix 1 Table 5. Oral Fluid RT-qPCR Cycle Threshold Values by Group and Day Post Inoculation*

Group	Day Post Inoculation						
	-4	0	1	2	3	4	28
D1.2/OR	40.0	NA	31.9	30.6	34.4	35.6	40.0
Control	40.0	40.0	NA	40.0	40.0	40.0	NA

*Blue highlighted values indicate a sample or tissue with a positive interpreted result by RT-qPCR per the manufacture's recommendations (Ct < 38). NA indicates sample not available.

Appendix 1 Table 6. Serology Results by Animal and Assay*

		NP IDEXX ELISA				H5 Serology			
Group	Animal ID	Pre-inoculation	5 DPI	7 DPI	35 DPI	Bald Eagle LPAIV H5 HI Titer	D1.2 HPAIV H5 HI Titer	D1.2 HPAIV SN Titer	IDVet H5 ELISA (%)
Control	652	0.93	0.86	-	-	ND	<1:10	<1:10	102.70
	653	1.06	0.89	-	-	ND	ND	ND	104.20
	654	0.97	0.93	-	-	ND	ND	ND	105.30
	641	1.21	0.69	-	-	<1:10	ND	ND	104.30
D1.2/OR	642	1.14	0.80	-	-	<1:10	ND	ND	104.30
	643	1.17	1.17	-	-	<1:10	ND	ND	102.90
	644	1.27	1.10	-	-	<1:10	ND	ND	100.90
	645	1.19	1.08	-	-	<1:10	ND	ND	104.00
	646	1.28	1.11	-	-	<1:10	ND	ND	107.30
	647	1.44	0.99	-	-	<1:10	ND	ND	110.90
	648	1.32	0.80	-	-	<1:10	ND	ND	101.20
	649	0.98	-	**0.43**	**0.39**	10	10	**40**	**20.30**
	650	0.96	-	**0.55**	**0.25**	**40**	10	**40**	**6.60**
	651	0.98	-	**0.50**	**0.31**	10	0	20	**20.00**

*A reciprocal HI or SN titer ≥ 40 was considered positive. A cutoff of < 0.6 was considered positive on NP IDEXX ELISA. A 38.5% cutoff was considered positive for the IDVet H5 ELISA. DPI indicates post inoculation. Bold indicates a positive test. -, indicates sample not taken. ND, Not done.

Appendix 1 Table 7. Influenza A virus Nucleoprotein Immunohistochemistry (IHC) Results by Tissue and Animal*

Cassette		D1.2/OR											Control		
ID	Tissue	641	642	643	644	645	646	647	648	649	650	651	652	653	654
B	Olfactory Bulb	+	-	-	-	-	-	-	-	-	-	-	-	ND	ND
D	Cerebrum	-	-	-	-	-	-	-	-	-	+	-	ND	ND	ND
E	Cerebrum	-	-	-	-	-	-	-	-	-	+	-	ND	ND	ND
F	Cerebrum	-	-	-	-	-	-	-	-	-	+	-	ND	ND	ND
G	Cerebrum	-	-	-	-	-	-	-	-	-	+	-	ND	ND	-
H	Cerebrum	-	-	-	-	-	-	-	-	-	-	-	ND	ND	ND
I	Cerebellum	-	-	-	-	-	-	-	-	-	-	-	ND	ND	ND
J	Cerebellum	-	-	-	-	-	-	-	-	†	-	-	ND	ND	ND
K	Lung	-	-	-	-	-	-	-	++	-	-	-	-	-	-
L	Lung	-	-	-	-	-	-	+	-	-	-	-	-	-	-
M	Lung	-	-	-	-	+	-	-	-	-	-	-	-	-	-
N	Spleen and Pancreas	-	-	ND	ND	ND	ND	ND	ND	ND	ND	ND	ND	ND	ND
P	TB LN	++	+	+	+	++	++	-	-	ND	-	-	-	-	-
Q	Trachea	-	-	+	-	+	-	-	-	-	-	-	-	-	-
R	Turbinate	+	-	-	-	++	-	ND	++	-	-	-	-	-	-
S	Ethmoid	++	++	+ +/-	++	-	++	++	++	+	++	ND	-	ND	-
T	Ileum	++	ND	ND	ND	-	ND	ND	-	ND	ND	ND	-	ND	ND
U	Spiral Colon	- (+)‡	ND	ND	ND	-	ND	ND	ND	-	ND	ND	ND	ND	ND
V	Diaphragm	-	ND	ND	ND	-	ND	ND	ND	ND	ND	ND	ND	ND	ND
W	Diaphragm	-	ND	ND	ND	+	ND	ND	ND	ND	ND	ND	ND	ND	-
X	Diaphragm	-	ND	ND	ND	-	ND	ND	ND	ND	ND	ND	ND	ND	-
Y	Diaphragm	-	ND	ND	ND	+	ND	ND	ND	ND	ND	ND	ND	ND	ND

*ND – Not done; IHC not performed due to study design, RT-qPCR results, or tissue was not available.
†Inconclusive staining results.
‡Animal 641 spiral colon stained negative for NP primary antibody, a lymph node associated with the apex of spiral colon that was found on the slide was positive.

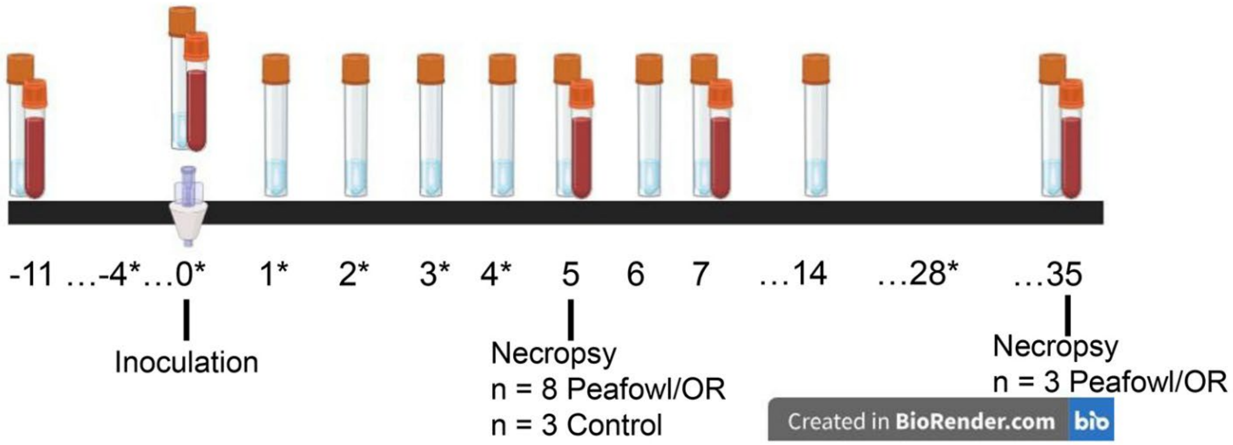

Appendix 1 Figure 1. Study Design. Swab icons indicate a nasal and fecal swab taken from all animals. Blood tubes represent MTM whole blood and serum taken. The three inoculated animals that were not necropsied on 5 days post inoculation (DPI) did not have blood taken on 5 DPI, instead blood was drawn from these animals on 7 DPI. Asterisks indicted days that cotton ropes were hung, and oral fluids were collected. Created in BioRender. Seger, H. (2026) https://BioRender.com/rm9fk7x.

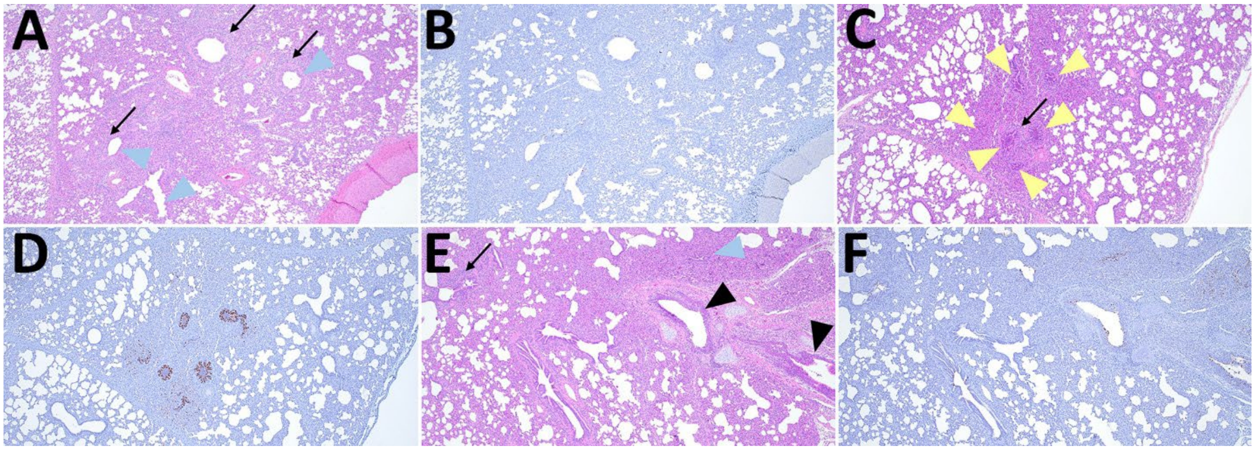

Appendix 1 Figure 2. Histologic lesions and detection of influenza A virus nucleoprotein (NP) in the lung. At 5 days post-inoculation, necrotizing bronchitis (black arrowheads), bronchiolitis (blue arrowheads), and purulent bronchiolitis (yellow arrowheads) and peribronchiolar cuffing (arrows) in animal 645 (A), 647 (C), and 648 (E) (40X). IAV NP antigen detected by IHC (brown) in the respiratory epithelium of effected conducting airways in animals 645 (B), 647 (D), and 648 (F) (40X).

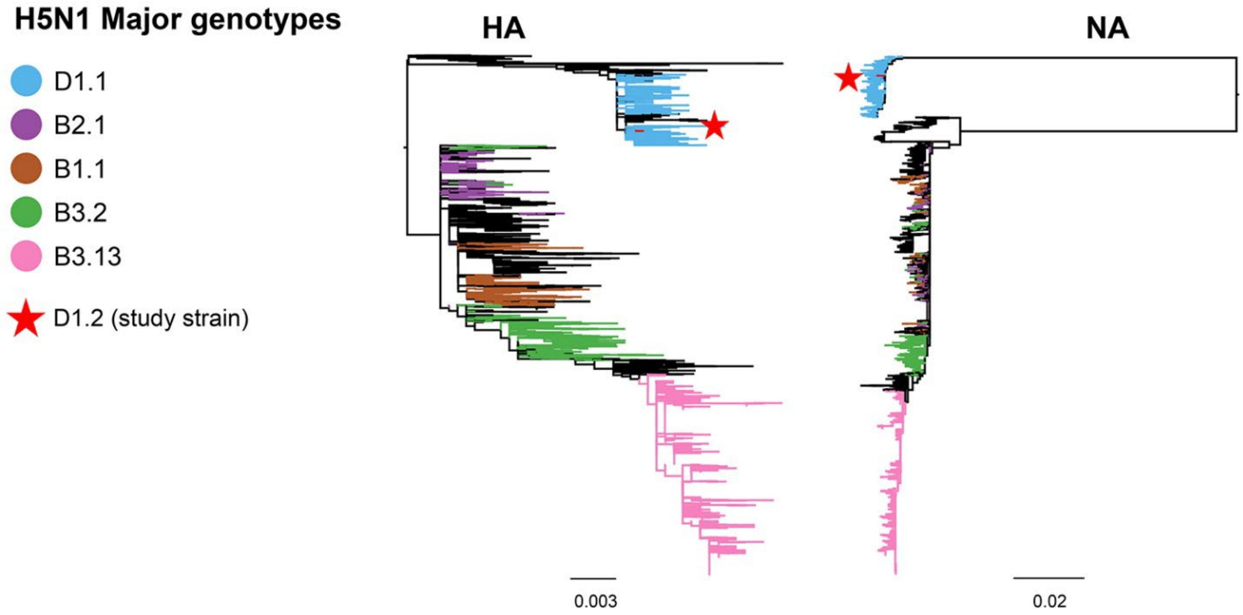

Appendix 1 Figure 3. HA and NA Phylogenetic Trees for HPAI H5N1 Strains Circulating in North America. The diversity of H5 clade 2.3.4.4b gene segments collected in North America 2022-present (left) and the associated N1 segments (right). The phylogenetic trees are colored respective to 5 most frequently detected genotypes. The D1.2 strains, including the A/D1.2/Oregon/24-031478-001/2024 study strain, are nested within the broader D1.1 clade on both trees and are highlighted in red.

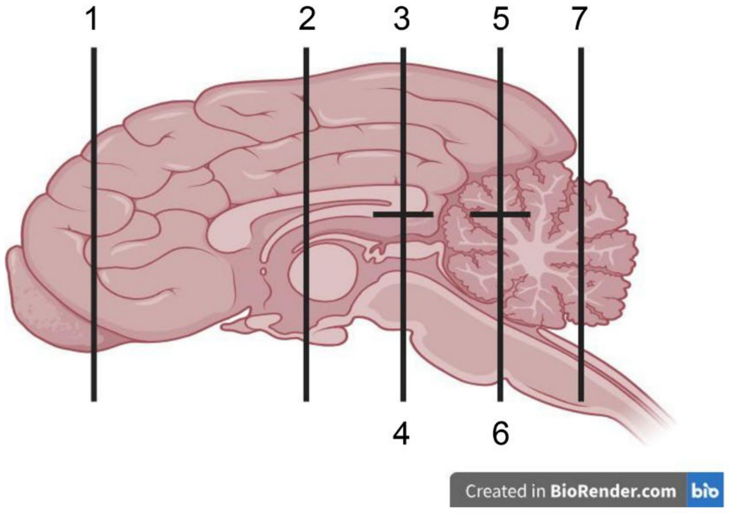

Appendix 1 Figure 4. Brain Sectioning. Representation of the 5 brain cross-sections taken on each animal, resulting in 7 tissue sections that were processed and evaluated by routine methods. Created in BioRender. Seger, H. (2026) https://BioRender.com/rgkgwx2.

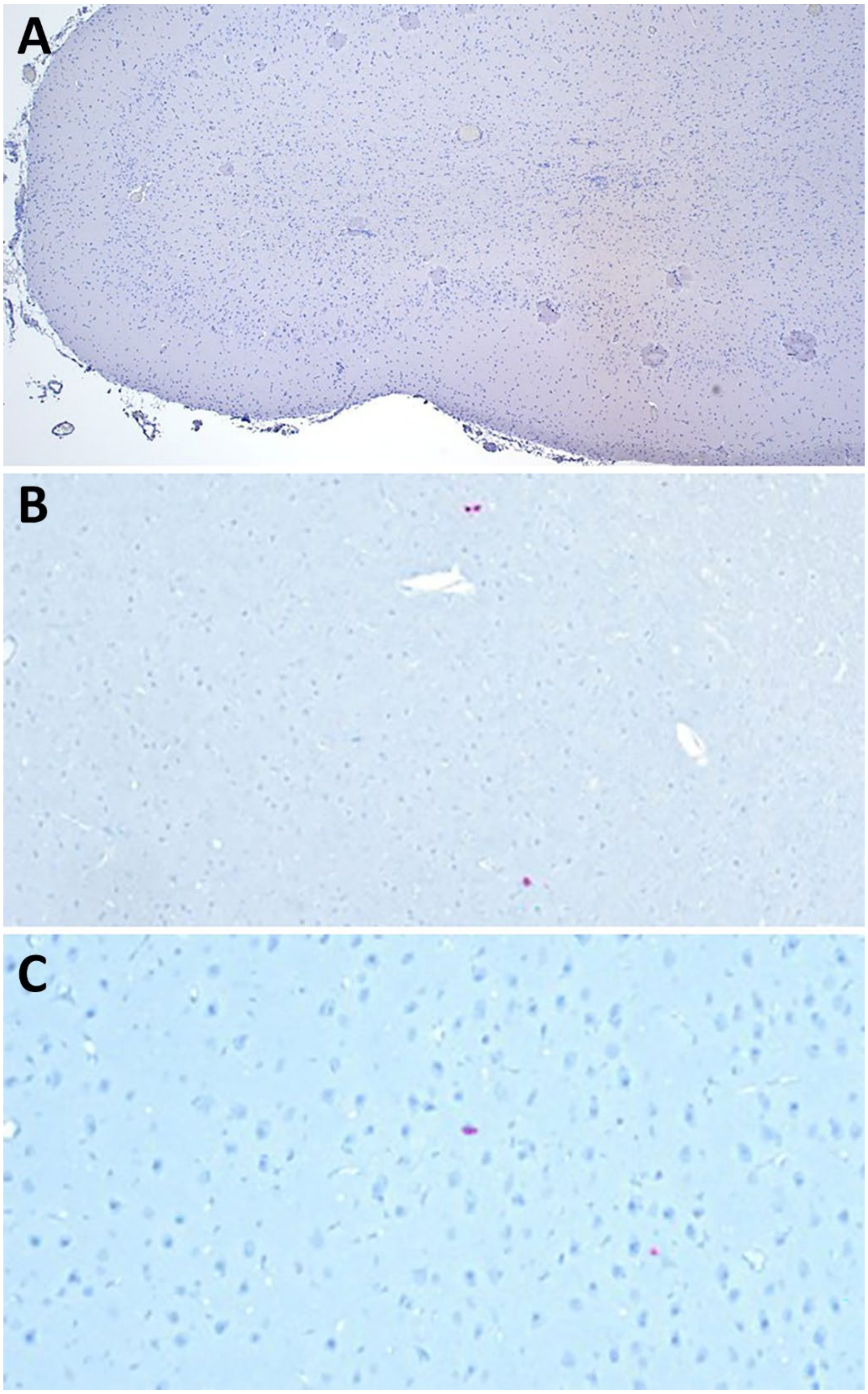

Appendix 1 Figure 5. In situ assays on cerebrum of Animal 650 at 35 days post inoculation. No influenza A virus (IAV) nucleoprotein (NP) antigen (brown) or non-specific staining in the same area with gliosis as Figure 1A with the exclusion of the primary antibody (A, animal 650; 40X). IAV NP replicating RNA (red) detected in occasional cells (B, animal 650; 100X). IAV NP non-replicating RNA detected in rare cells (C, animal 650; 200X).
